# Two cases of unresectable hepatocellular carcinoma treated via atezolizumab and bevacizumab combination therapy

**DOI:** 10.1186/s40792-023-01678-9

**Published:** 2023-06-02

**Authors:** Ryosuke Tsunemitsu, Motoyasu Tabuchi, Shinya Sakamoto, Kenta Ogi, Manabu Matsumoto, Jun Iwata, Takehiro Okabayashi

**Affiliations:** 1 Department of Gastroenterological Surgery, Kochi Health Sciences Center, 2125-1 Ike, Kochi-City, Kochi 781-8555 Japan; 2 Department of Radiology, Kochi Health Sciences Center, 2125-1 Ike, Kochi-City, Kochi 781-8555 Japan; 3 Department of Diagnostic Pathology, Kochi Health Sciences Center, 2125-1 Ike, Kochi-City, Kochi 781-8555 Japan

**Keywords:** Advanced hepatocellular carcinoma, Conversion surgery, Atezolizumab plus bevacizumab

## Abstract

**Background:**

Treatment of hepatocellular carcinoma (HCC) varies widely depending on the patient's condition. In recent years, combination therapy with immune checkpoint inhibitors has emerged as the treatment of choice due to its superior antitumor effects for unresectable HCC (uHCC). Conversion surgery (CS) after systemic chemotherapy is expected to be an effective treatment strategy for uHCC. Here, we report two cases of uHCC with bilateral porta hepatis invasion, in which atezolizumab plus bevacizumab therapy regressed the tumor invasion of the porta hepatis, followed by CS with R0 resection.

**Case presentation:**

The first patient—a 71-year-old man with S4 HCC—developed porta hepatis, and the tumor compressed the right portal vein and bile duct. R0 resection with left trihepatectomy was impossible because of insufficient liver function, and combination therapy using atezolizumab and bevacizumab was initiated. After ten courses of treatment, the tumor shrunk with regression of the porta hepatis contact, and segmentectomy of S4 was performed with a sufficient surgical margin. Histopathological findings showed that the primary tumor was mostly necrotic with no residual viable tumor cells. The second patient was a 72-year-old man with an S4 HCC extending to the porta hepatis. The patient’s condition was almost similar to that in the first case and required left tri-segmentectomy with R0 resection; however, insufficient liver function made liver resection impossible. An atezolizumab plus bevacizumab regimen was administered, and after seven courses of treatment, porta hepatis compression regressed, following which left lobectomy was performed with adequate surgical margins. The pathological diagnosis was moderately differentiated HCC, most of which was necrotic, and R0 resection was confirmed.

**Conclusions:**

Atezolizumab plus bevacizumab therapy has the potential to facilitate radical resection in patients with uHCC.

## Introduction

Hepatocellular carcinoma (HCC) is the sixth most common cancer worldwide and the second most common cause of cancer-related death [1]. Approximately half of the patients with HCC will ultimately be treated with systemic therapies, and the prognosis will remain poor, although surgical resection and locoregional therapy are the most effective treatments for HCC [2]. In addition, HCC frequently occurs in elderly patients with impaired physical conditions. This is because HCC often occurs in functionally compromised livers, where pharmacological tumor therapy is challenging. Until 2020, the treatment of unresectable HCC (uHCC) has been insufficient; the onset of immunotherapy has shed light on the prognosis of patients with uHCC. Atezolizumab combined with bevacizumab therapy in patients with uHCC was reported to be superior to sorafenib therapy in terms of overall survival (OS) and progression-free survival in the IMbrave150 trial [3, 4].

Recently, a new treatment strategy, called atezolizumab–bevacizumab curative (ABC) conversion, has been proposed, wherein curative treatments, such as hepatic resection, radiofrequency ablation, and superselective transcatheter arterial chemoembolization, are performed after achieving marked tumor shrinkage with atezolizumab–bevacizumab combination therapy [5]. Patients with intermediate-stage HCC (locally advanced HCC without vascular invasion or extrahepatic spread) are the primary candidates for ABC conversion therapy. Herein, we report the clinical course of two cases of uHCC curatively treated with conversion surgery following shrinkage of uHCC with a combination therapy of atezolizumab plus bevacizumab.

## Case presentation

### Case 1

The patient was a 71-year-old man with a history of chronic alcoholic liver disease, hypertension, dyslipidemia, diabetes, and asthma. In March 2021, during routine follow-up, a medical examination revealed liver dysfunction, and computed tomography (CT) showed a large liver tumor. The patient was referred to our department for further diagnosis and management.

Blood tests performed at his first visit revealed an elevated alanine aminotransferase level of 75 U/L, aspartate aminotransferase level of 51 IU/L, and total bilirubin level of 1.7 mg/dL. Albumin level (4.2 d/dL) and prothrombin time level (84.6%) were normal. Serum alpha-fetoprotein (AFP) level was 106.6 ng/mL, and that of des-gamma carboxyprothrombin (DCP) was 67,200 mAU/mL. He had a Child–Pugh score of 5 (Child–Pugh grade A) and no hepatic encephalopathy or ascites (Table 1). Contrast-enhanced CT showed an internally enhanced 120-mm tumor in the S4 area of the liver, which touched the right hepatic hilum, compressing the right portal vein and bile duct. Intrahepatic dilation of the lateral and anterior segments of the liver was also observed (Fig. 2A). The patient was diagnosed with Barcelona Clinic Liver Cancer stage A HCC, but the tumor was extensive in the right lobe, and left tri-segmentectomy was required to achieve radical resection. Remnant liver function was assessed using remnant (rem) KICG (= KICG × volume rate) and rem 99mTc–GSA scintigraphy (KGSA) (= KGSA × functional rate) indices; hepatectomy was considered unsafe for values < 0.05. The patient’s liver volume was 1053 ml, and effective liver resection rate of 65.3%. The remnant liver volume was 365 ml, and the remnant liver function was 0.048521 in rem KICG and 0.055739 in rem KGSA, considering it unresectable because of the difficulty in maintaining remnant liver function after tri-segmentectomy of the liver.

**Table 1:** Laboratory investigation in Case 1

Laboratory examination							
*Complete blood cell count*			γGTP	1518	U/L	*Viral markers*	
WBC	4350	u/L	ChE	319	U/L	HBs antigen	(–)
RBC	448	10^4^ u/L	Na	140	mEg/L	HCV antibody	(–)
Hg	15	g/dL	K	4.3	mEg/L	*Liver function*	
Ht	46.2	%	Cl	104	mEg/L	ICG-R	21.3%
Plt	15.5	10^4^ u/L	TP	7.2	g/dL	Child–Pugh score	5
*Blood coagulation test*			Alb	4.2	g/dL	Child–Pugh grade	A
PT (%)	84.6	%	BUN	13.1	mg/dL	ALBI score	-2.19
PT-INR	1.11		Cr	0.76	mg/dL	mALBI grade	2b
*Blood chemistry*			CRP	0.24	mg/dL		
T-Bil	1.7	mg/dL	HbA1c	6	%		
AST	75	U/L	*Tumor markers*				
ALT	51	U/L	AFP	160.6	ng/mL		
LDH	259	U/L	DCP	67,200	mAU/mL		
ALP	466	U/L					

γGTP: γ-glutamil transpeptidase, AFP: alpha-fetoprotein, Alb: albumin, ALBI: albumin–bilirubin, ALP: alkaline phosphatase, ALT: alanine aminotransferase, AST: aspatate aminotransferase, BUN: blood–urea–nitrogen, ChE: cholinesterase, Cr: creatinine, CRP: C-reactive protein, DCP: des-gamma carboxyprothrombin, Hb: hemoglobin, HbA1c: hemoglobin A1c, HBs antigen: hepatitis B surface antigen, HCV antibody: hepatitis C virus antibody, Ht: hematocrit, ICG-R: indocyanine green retention, LHD: lactate dehydrogenase, Plt: platelets, PT: prothrombin time, PT-INR: international normalized ratio of prothrombin time, RBC: red bllod cells, T-Bil: total bilirubin, TP: total protein, WBC: white blood cells

**Fig. 1:**
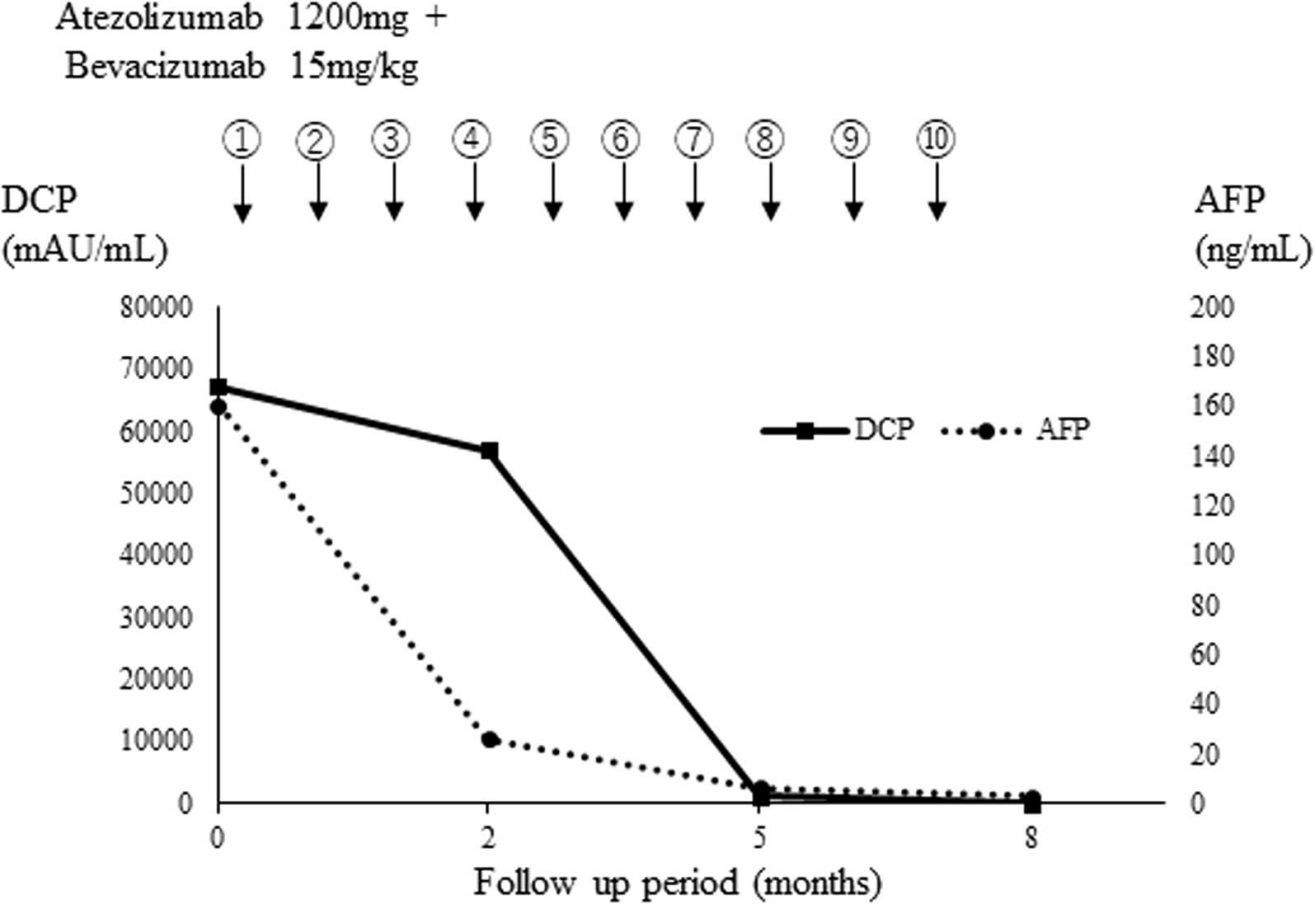
Time line of chemotherapy and changes in levels of DCP and AFP in case 1. The circle numbers are administration courses. *AFP* alph-fetoprotein, *DCP* des-gamma carboxyprothrombin

**Fig. 2:**
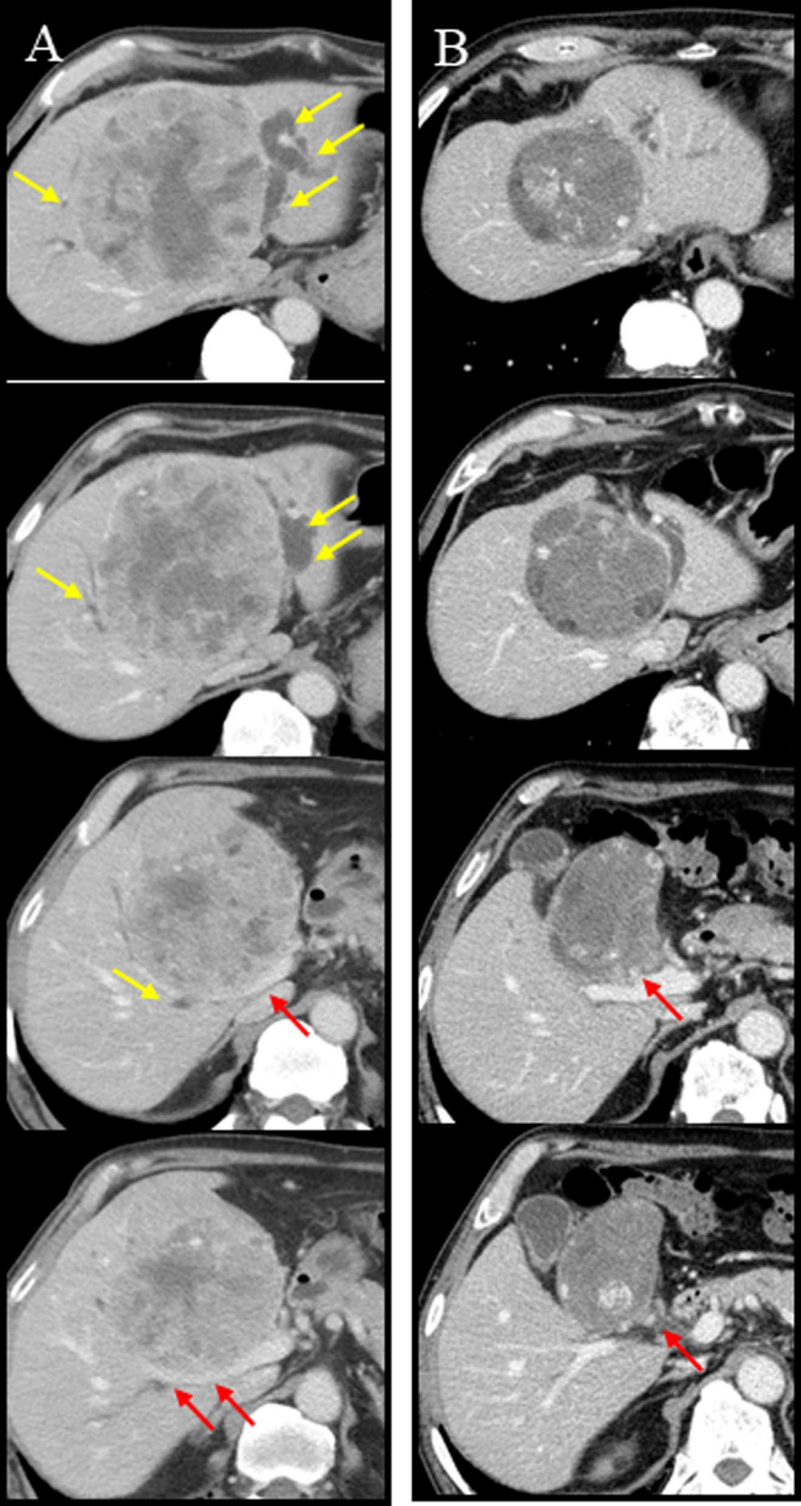
Computed tomography of case 1. **A** Neoplastic lesion in S4 at the time of diagnosis. Yellow arrows show dilatation of intrahepatic bile duct in anterior, and lateral segment. Red arrows show tumor contact with right hilum region. **B** Neoplastic lesion in S4 after course of ATEZO/BEV. Tumor contact with hilum region regressed to only left hilum (red allows)

According to the fourth edition in 2020, including the 2017 edition of the Japanese Society of Hepatology Guidelines, atezolizumab (1200 mg) plus bevacizumab (15 mg/kg) was administered as one course over 3 weeks without any adverse effects. After the tenth course, the serum levels of AFP and DCP were within the normal range (Fig. 1), the intrahepatic HCC had shrunk to 73 mm with regression of the right hepatic hilum tumor contact (Fig. 2B), and the patient was diagnosed with a partial response to the modified RECIST. R0 resection with left hemihepatectomy is considered feasible with a sufficient surgical margin. In the left hemihepatectomy, the remnant liver volume was 78.4% and rem KICG was 0.077616, and the remnant liver function was 0.084348 in rem KICG, and 0.10263192 in KGSA.

**Fig. 3:**
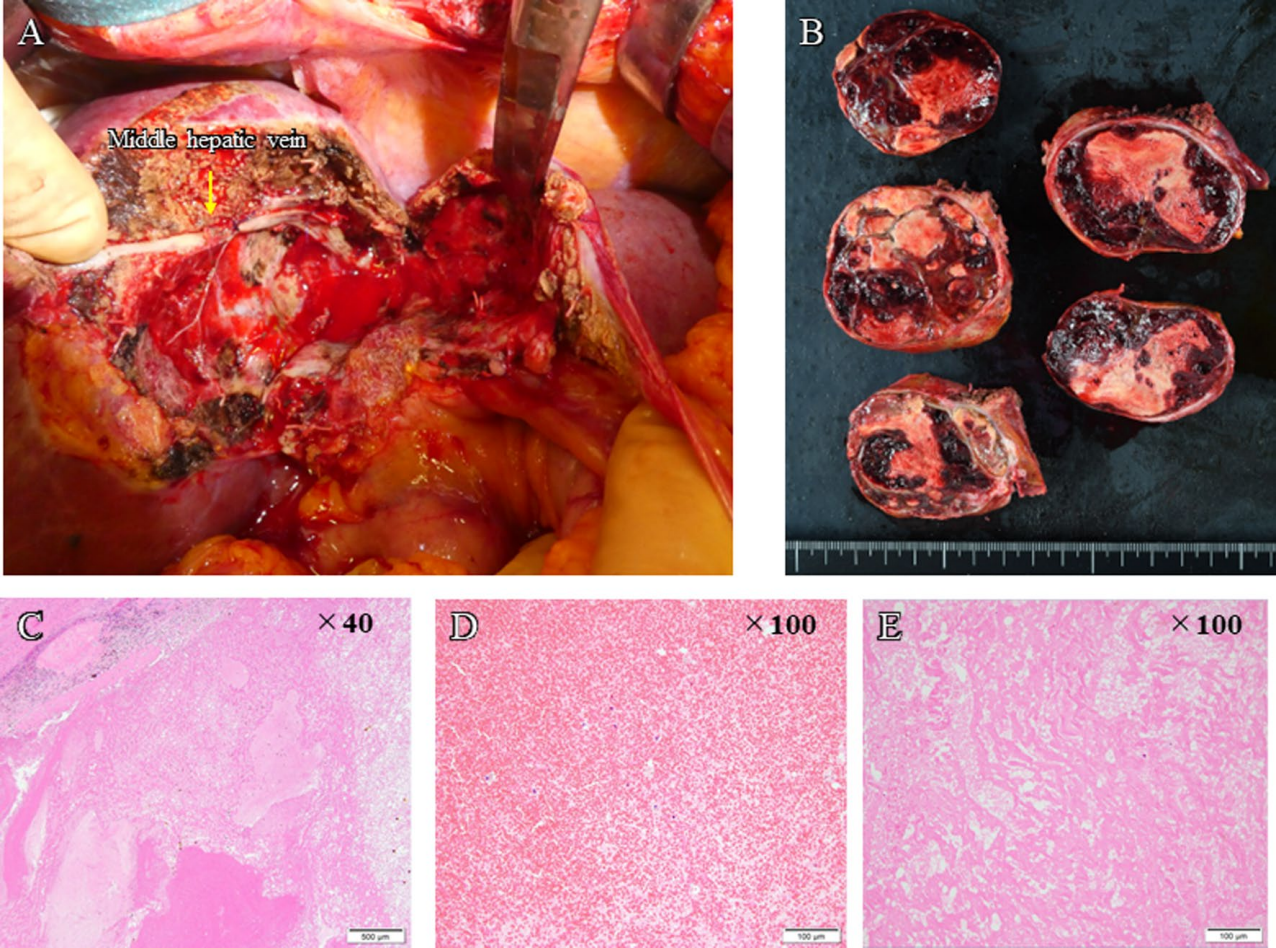
**A** Surgical findings. **B** Resection specimen obtained after conversion surgery. **C–E** Hematoxylin and eosin (H&E) stain. No viable hepatocellular carcinoma was detected

In November 2021, segmentectomy of S4 and cholecystectomy were performed following a 5-week period since the last administration of ATZ + BEV. The duration of surgery was 4 h 13 min; blood loss was 1280 mL, and intraoperative and postoperative blood transfusions (4 units of fresh frozen plasma) were required. Intraoperative ultrasonography was used to confirm the location of the tumor, and sufficient surgical margin was obtained. The pathological diagnosis was necrosis without viable HCC cells. There was only a small amount of background liver tissue and little or no invasion into the portal vein. The resection margins were negative and R0 resection was confirmed (Fig. 3). The patient was discharged 10 days after the surgery without postoperative complications. There was no recurrence within one year of surgery.

### Case 2

The patient was a 72-year-old man who visited his family doctor complaining of upper abdominal pain and was referred to our department after a liver tumor was detected on abdominal ultrasonography. The patient had no medical history of viral hepatitis or metabolic disorders.

Blood tests at his first visit revealed an elevated alanine aminotransferase level of 254 U/L, an aspartate aminotransferase level of 230 IU/L, and a total bilirubin level of 3.7 mg/dL. Albumin level (3.9 d/dL) and prothrombin time level (90.8%) were normal. The serum level of alpha-fetoprotein (AFP) was 570.6 ng/mL and that of des-gamma carboxyprothrombin (DCP) was 65,143 mAU/ml. He had a Child–Pugh score of 6 (Child–Pugh grade A) and no hepatic encephalopathy or ascites (Table 2). Contrast-enhanced CT showed an internally enhanced 120-mm tumor in the S4 area of the liver, which was in contact with the right hilum region, and intrahepatic dilation of the left and anterior segments of the liver (Fig. 5A).

**Table 2:** Laboratory investigation in Case 2

Laboratory examinations							
*Complete blood cell count*			γGTP	547	U/L	*Viral markers*	
WBC	5510	u/L	ChE	219	U/L	HBs antigen	(–)
RBC	389	10^4^ u/L	Na	139	mEg/L	HCV antibody	(–)
Hg	12.8	g/dL	K	3.6	mEg/L	*Liver function*	
Ht	39.5	%	Cl	103	mEg/L	ICG-R	26.6%
Plt	12.6	10^4^ u/L	TP	7.6	g/dL	Child–Pugh score	6
*Blood coagulation test*			Alb	3.9	g/dL	Child–Pugh grade	A
PT (%)	90.8	%	BUN	17.3	mg/dL	ALBI score	-1.92
PT-INR	1.05		Cr	0.61	mg/dL	mALBI grade	2b
*Blood chemistry*			CRP	0.64	mg/dL		
T-Bil	3.7	mg/dL	HbA1c	6	%		
AST	254	U/L	*Tumor markers*				
ALT	230	U/L	AFP	570.6	ng/mL		
LDH	447	U/L	DCP	65,143	mAU/mL		

γGTP: γ-glutamil transpeptidase, AFP: alpha-fetoprotein, Alb: albumin, ALBI: albumin–bilirubin, ALP: alkaline phosphatase, ALT: alanine aminotransferase, AST: aspatate aminotransferase, BUN: blood–urea–nitrogen, ChE: cholinesterase, Cr: creatinine, CRP: C-reactive protein, DCP: des-gamma carboxyprothrombin, Hb: hemoglobin, HbA1c: hemoglobin A1c, HBs antigen: hepatitis B surface antigen, HCV antibody: hepatitis C virus antibody, Ht: hematocrit, ICG-R: indocyanine green retention, LHD: lactate dehydrogenase, Plt: platelets, PT: prothrombin time, PT-INR: international normalized ratio of prothrombin time, RBC: red bllod cells, T-Bil: total bilirubin, TP: total protein, WBC: white blood cells

**Fig. 4:**
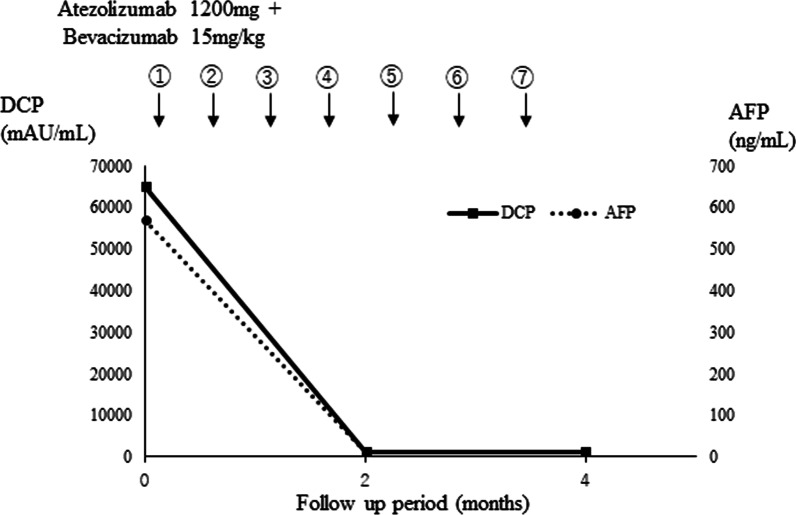
Time line of chemotherapy and changes in levels of DCP and AFP in case 2. The circle numbers are administration courses. *AFP*  alph-fetoprotein, *DCP*  des-gamma carboxyprothrombin

**Fig. 5:**
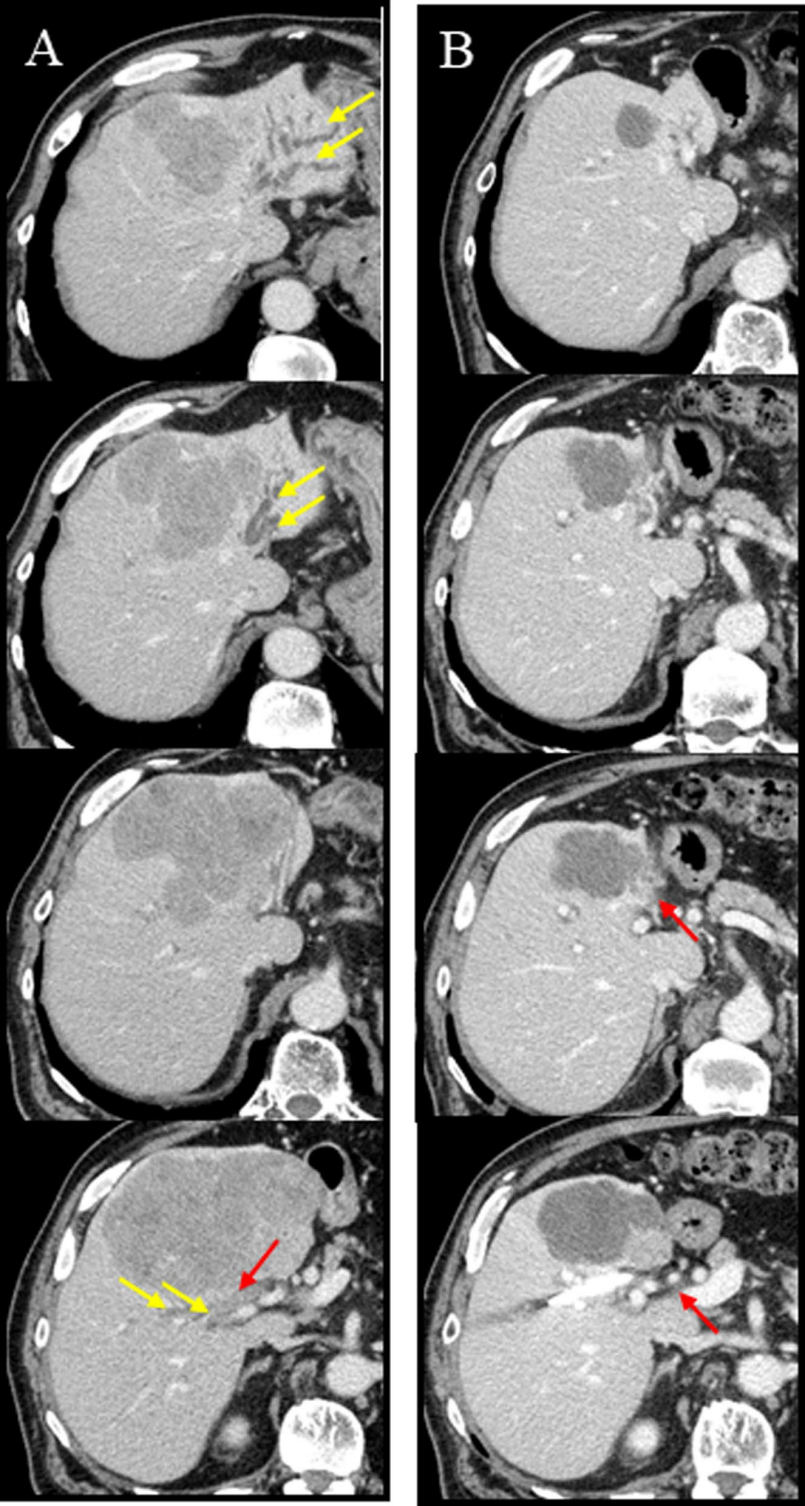
Computed tomography of case 2. **A** Neoplastic lesion in S4 at the time of diagnosis. Yellow arrows show dilatation of intrahepatic bile duct in anterior, and lateral segment. Red arrows show tumor contact with right hilum region. **B** Neoplastic lesion in S4 after course of ATEZO/BEV. Tumor contact with hilum region regressed (red allows)

The patient was diagnosed with Barcelona Clinic Liver Cancer stage A HCC, accompanied by right hepatic hilum involvement. As in Case 1, left tri-segmentectomy was needed to achieve radical resection, but the remnant liver volume was 21.1% (410 ml) and rem KICG was 0.02235 after tri-segmentectomy, indicating surgical resection to be impossible. Endoscopic retrograde cholangiopancreatography was performed to relieve jaundice, and atezolizumab (1200 mg) and bevacizumab (15 mg/kg) were administered as one course of 3 weeks without any adverse effects. After the seventh course, the serum levels of AFP and DCP were within the normal range (Fig. 4), the intrahepatic HCC had shrunk to 70 mm with regression of the hilum tumor contact, and the patient was diagnosed with a partial response to the modified RECIST (Fig. 5B). R0 resection with left hemihepatectomy is considered feasible with a sufficient surgical margin. With left hemihepatectomy, the remnant liver volume was 65.5% (1275 ml) and rem KICG was 0.06943, and the remnant liver function was 0.10263912 in rem KICG and 0.077616 in KGSA.

**Fig. 6:**
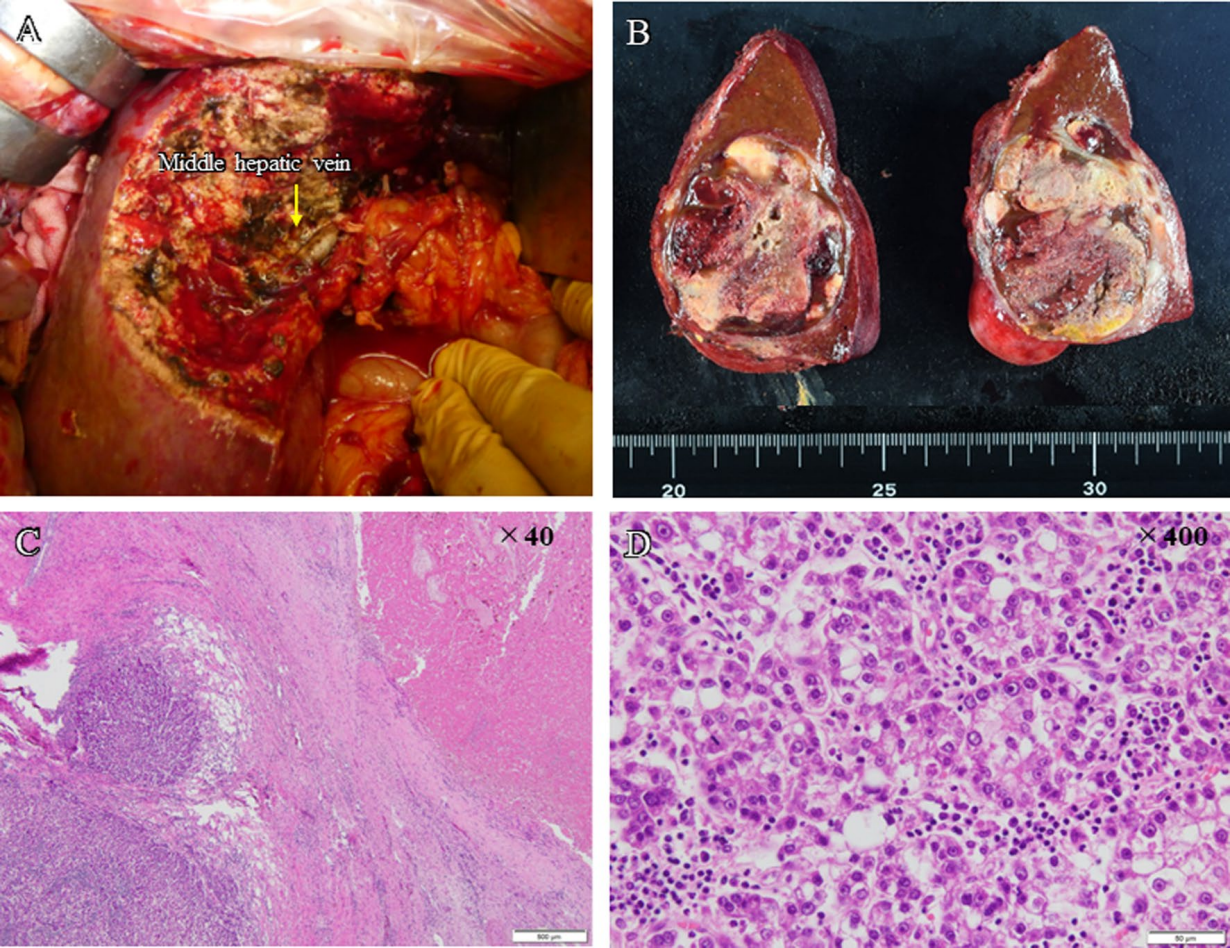
**A** Surgical findings. **B** Resection specimen obtained after conversion surgery. **C**, **D** Hematoxylin and eosin (H&E) stain. A primary tumor composed of moderately differentiated cancer cells with most of these tissues necrotic

In December 2022, a left hepatectomy was performed after a 5-week period following the last ATZ + BEV administration. The duration of surgery was 4 h 16 min; blood loss was 1810 ml, and intraoperative and postoperative blood transfusions (red blood cells, 4 units; fresh frozen plasma, 4 units; and blood platelets, 10 units) were performed. Intraoperative ultrasonography confirmed the absence of tumor invasion into hepatic hilum, and sufficient surgical margin was obtained. The pathological diagnosis was moderately differentiated HCC, with most of the tissues being necrotic, possibly because of preoperative treatment. Although the tumor tissues were present outside the capsule, the margins were negative, and R0 resection was confirmed (Fig. 6). The patient was discharged 8 days after surgery without postoperative complications. There was no recurrence up to 5 months after surgery.

## Discussion

We report two cases of uHCC in which atezolizumab plus bevacizumab therapy resulted in regression of uHCC tumor. To our knowledge, this is the first report of technically unresectable tumors due to insufficient remnant liver function that successfully underwent conversion surgery with R0 resection. Regarding conversion surgery for ATZ/BEV, we found seven case reports including our two cases (Table3) [6–11]. Among the eight patients, five had advanced stage HCC with distant metastasis or portal vein tumor thrombosis (PVTT), one had intermediated-stage HCC previously treated with transcatheter arterial chemoembolization (TACE). In the background liver, four patients had non-viral HCC and one patient had HVB-related HCC. All eight patients, including our patients, achieved both cancer free and drug-free status following conversion surgery.

**Table 3:** Literature review

References	Year	Age	Gender	Etiology	BCLC	Unresectable reason	Size maximum (cm)		Regimen	Cycles	Pathological	f/u	Cancer-free/drug free
							Pre	Post			Response	(month)	
[6]	2021	68	M	Non-viral	C	Rt adrenal meta	72	ND	ATZ/BEV → LEN	4	PR	5	+ / +
[7]	2021	63	M	ND	B	Tumor spread bilateral lobes	15.5	17.2	TACE → ATZ/BEV	15	PR	19	+ / +
[8]	2022	79	M	ND	C	PVTT	ND	ND	ATZ/BEV + TAE	2	PR	5	+ / +
[9]	2022	77	M	HBV	C	Rt adrenal meta, IVC invasion	15.2	9.9	ATZ/BEV	9	CR	3	+ / +
[10]	2022	77	M	Non-viral	C	Lung meta	16.8	12.7	ATZ/BEV	7	CR	8	+ / +
[11]	2023	75	M	ND	C	Peritoneal metastasis	5.7	4.7	ATZ/BEV + TAE	15	CR	7	+ / +
Our case	2023	71	M	Non-viral	A	Remnant liver volume	12.5	7.3	ATZ/BEV	10	CR	12	+ / +
Our case	2023	72	M	Non-viral	A	Remnant liver volume	11.6	6.4	ATZ/BEV	7	PR	5	+ / +

BCLC Barcelona Clinic Liver Cancer grade, ATZ/BEV atezolizumab and bevacizumab combination therapy, LEN lenvatinib, HBV hepatitis B virus, TACE transcatheter arterial chemoembolization, TAE transcatheter arterial embolization, PVTT portal vein tumor thrombosis, IVC inferior vena cava, ND no data

Surgical hepatic resection is the mainstay of curative treatment for patients who have good functional liver reserve [12, 13]. This might be the same issue in patients who achieved remarkable tumor shrinkage with atezolizumab plus bevacizumab combination therapy. In recent years, several new agents with excellent anticancer effects have been approved for systemic therapy, and the use of conversion surgery for uHCC is expected to increase [14–16]. Preventing recurrence after curative surgical treatment of advanced-stage HCC remains a major challenge, and it has been suggested that even radiologically assessed resectable advanced-stage HCC is a systemic disease. Thus, the neoadjuvant setting for uHCC may be effective in determining tumor response and assessing in vivo sensitivity. However, it is important to note that neoadjuvant therapy may not completely eliminate micrometastases that are not detected during imaging, unless unnecessary surgical treatment can be avoided if distant metastasis or disseminated disease appears during preoperative neoadjuvant therapy.

The current major questions include how background liver etiology influences the treatment efficacy in neoadjuvant setting, which lenbatinib, sorafenib, or ATZ/BEV should be used in the neoadjuvant setting with conversion surgery in mind, how surgical indications for patients with uHCC impact the overall survival after curative surgical management with atezolizumab plus bevacizumab therapy, accepting the proven impact on both local and distant metastatic recurrences, and whether new antitumor agents in the adjuvant setting improve outcomes. Pfister et al. demonstrated through preclinical models of NASH-induced HCC that the increase of hepatic CD8^+^PD1^+^ T cells induced by immunotherapy impairs immune surveillance and did not lead to tumor regression [17]. In our case, although both patients had non-viral HCC, their resected liver specimens only showed very mild fatty liver (0–1%), which was not indicative of NASH. We speculate that the absence of an inflammatory microenvironment and immune exhaustion due to fatty liver in our cases might have contributed to the significant response to immunotherapy. The optimal regimen to facilitate CS is controversial, reported CS rate after Lenvatinib treatment was 1.3–15.0%, whereas the antitumor effect of sorafenib was weak and the success of subsequent CS was low [18, 19]. Further studies are needed to clarify the optimal regimen for conducting CS. Advanced-stage HCC patients who are candidates for surgical resection should undergo an assessment of early recurrence risk to avoid unnecessary and ineffective resection, as well as to identify patients for whom palliative or alternative treatment may be the treatment of choice, even if the surgeons consider uHCC to be completely removed based on preoperative imaging after atezolizumab plus bevacizumab combination therapy. In the cases reported herein, no sign of recurrence was observed in the two patients. Since the effect of atezolizumab plus bevacizumab therapy was assessed based on preoperative treatment, we believe that it is not too late to use anticancer agents after recurrence. The HCC community throughout the world is curiously waiting to see the directions of this study about the adjuvant setting in patients with uHCC who underwent conversion surgery, which will help understand the details of the study design, setting, participants, study methodology used, outcome measures, and the results and their relevance to patients with uHCC.

## Conclusion

We describe two cases of uHCC that were successfully treated with conversion surgery after atezolizumab plus bevacizumab combination therapy. This therapeutic approach may improve the outcomes of patients with uHCC. As there is no consensus among experts on appropriate decision-making, we must continue to endeavor to improve outcomes in patients with uHCC. More data, including epidemiological and pathological findings, will be required to determine the appropriate surgical indications after atezolizumab plus bevacizumab therapy and the adjuvant setting for uHCC.

## Data Availability

All data generated or analyzed during this study are included in the published article.
